# Inverse Association of Serum Folate Level with Metabolic Syndrome and Its Components in Korean Premenopausal Women: Findings of the 2016–2018 Korean National Health Nutrition Examination Survey

**DOI:** 10.3390/nu14040880

**Published:** 2022-02-19

**Authors:** Ye-Seul Koo, Yong-Jae Lee, Jae-Min Park

**Affiliations:** 1 Department of Family Medicine, Gangnam Severance Hospital, Yonsei University College of Medicine, 211 Eonju-ro, Gangnam-gu, Seoul 06273, Korea; rndptmfrnd@yuhs.ac (Y.-S.K.); ukyjhome@yuhs.ac (Y.-J.L.); 2 Department of Medicine, Graduate School of Medicine, Yonsei University, 50-1 Yonsei-ro, Seodaemun-gu, Seoul 03722, Korea

**Keywords:** folate, metabolic syndrome, premenopause, women

## Abstract

Research on the association of serum folate levels with metabolic syndrome (MetS) in premenopausal women is lacking. This study was aimed to investigate this association in 1730 premenopausal women using the 2016 to 2018 Korean National Health and Nutrition Examination Survey data. Participants’ mean age and BMI were 35.9 years and 22.7 kg/m^2^, respectively. Participants were divided into three groups according to serum folate tertiles. Odds ratios (OR) and 95% confidence intervals (CIs) for abdominal obesity, elevated blood pressure (BP), high fasting plasma glucose (FPG), high triglycerides (TG), low high-density lipoprotein cholesterol (HDL-C), and MetS were calculated in multiple logistic regression models adjusted for possible confounders, by serum folate level tertiles. Prevalence of MetS (14.9, 11.0, and 8.6%); abdominal obesity (17.8, 16.0, and 11.4%); high TG (17.5, 14.0, and 11.1%); and low HDL-C (50.3, 44.6, and 42.5%) decreased with increasing folate level tertile. Prevalence of elevated BP (14.3, 12.0, and 11.7%) and high FPG (11.9, 15.8, and 13.0%) showed no significant differences according to serum folate level tertiles. The multivariate-adjusted ORs (95% CIs) for MetS, abdominal obesity, elevated BP, high TG, and low HDL-C in the highest folate level tertile were 2.17 (1.46–3.22), 1.80 (1.25–2.60), 1.77 (1.16–2.70), 1.90 (1.35–2.67), and 1.49 (1.14–1.94), respectively. The ORs for high FPG did not show significant differences according to serum folate level tertiles. In conclusion, serum folate levels were inversely associated with an increased risk of MetS in Korean premenopausal women. These results suggest that MetS can be prevented and managed by improving the serum folate levels in premenopausal women.

## 1. Introduction

Metabolic syndrome (MetS), characterized by a cluster of metabolic abnormalities such as abdominal obesity, elevated blood pressure (BP), high blood sugar, high triglycerides (TG), and low high-density lipoprotein cholesterol (HDL-C), is thought to be linked to the development of diverse medical disorders [1]. Individuals with MetS are more susceptible to cardiovascular diseases, type 2 diabetes mellitus, and certain aggressive cancers, which are the major causes of mortality worldwide [2,3,4,5]. With the spread of the westernized lifestyle, MetS has become a global epidemic, threatening public health [6].

While the prevalence of MetS in premenopausal women is lower than that in postmenopausal women, metabolic abnormalities in premenopausal women can progress during menopausal transition [7]. Moreover, national surveys from various countries have revealed greater weight increases in young adult women in recent years than in older women [8]. Thus, the prevention and management of MetS in premenopausal women is important from a public health perspective. Although the pathogenesis of MetS has not been fully clarified, it is believed to involve the interplay of genetic, environmental, and nutritional factors (including micronutrients) [9,10,11]. Since it is possible to manage most environmental and nutritional factors, appropriately controlling them can contribute to the prevention and management of MetS.

Folate, a water-soluble vitamin, also known as vitamin B9, is present in various natural foods such as legumes, green leafy vegetables, organ meats, and sprouts [12]. As this vitamin cannot be synthesized naturally by the human body, it must be acquired either through diet or supplementation [13]. Diets low in fresh vegetables, legumes, and fruits are related to folate deficiency. Besides unhealthy diets, alcoholism, certain medications, some genetic disorders, and various diseases can contribute to folate deficiency [14]. Low serum folate levels have been inculpated in neural tube defects [15]. There are concerns that excessive folate intake has the potential for hiding a vitamin B12 deficiency due to its inhibition of the development of anemia [16]. Generally, the recommended intake of folate for adults is 400 μg/day and serum folate levels of less than 3 ng/mL are considered as a folate deficiency status [17,18]. In European countries, studies revealed that most populations did not meet the recommended dietary folate intake levels, and that the average serum folate levels ranged from 2.8 ng/mL to 8.9 ng/mL [19,20]. In the United States, fortification of enriched cereal-grain products with folic acid has been mandatory since 1998. Median serum folate concentrations more than doubled from 5.5 ng/mL (in 1988–1994, pre-fortification) to 12.2 ng/mL (in 2005–2000, post-fortification) in the United States [21]. The World Health Organization reported that reproductive age women and children are at the highest risk of some micronutrient deficiency and some studies indicated that young adults are at risk of micronutrient deficiency owing to their unhealthy nutritional habits such as low consumption of vegetables and fruits [22,23,24]. Chen et al. revealed that young adults aged 19–30 years had the highest prevalence of folate deficiency and lowest mean serum folate levels among all age groups [24].

Previous studies revealed an association of lower serum folate levels with an increased risk of neural tube defects, certain cancers, and cardiovascular mortality [14,25,26,27]. In addition, some studies have indicated that epigenetic alterations, especially DNA methylation, may play an essential function in the pathogenesis of insulin resistance [28,29,30]. Folate, a crucial source of the one-carbon group utilized in methylation reactions and RNA/DNA synthesis, was found to be related to insulin resistance in nondiabetic adults in the United States [31]. Moreover, higher folate intake has been linked with better metabolic parameters in overweight/obese older adults with MetS [32]. Furthermore, increased body mass index (BMI), absolute amount of fat, and percent body fat are significantly related to lower serum folate concentrations in postmenopausal women [33]. However, research on the association between serum folate levels and MetS and its components in premenopausal women is lacking. Thus, we aimed to investigate this association in a nationally-representative sample of premenopausal Korean women.

## 2. Materials and Methods

### 2.1. Survey Overview and Study Population

This study used the 2016 to 2018 Korea National Health and Nutrition Examination Survey (KNHANES) data collected by the Korea Disease Control and Prevention Agency (KDCA). KNHANES is a representative, nationwide, population-based survey conducted to assess the health and nutritional status of South Koreans. Detailed information about the study population and methodology of KNHANES was reported in previous research [34].

The 24,269 participants that were included in the 2016 to 2018 KNHANES included 10,732 female adults aged ≥20 years. Of the 10,732 female adults, folate levels were examined in 3500 participants. Of the 3500 participants who had available serum folate level data, we excluded pregnant women, postmenopausal women, and participants who underwent hysterectomy and/or oophorectomy (*n* = 1667). Of the remaining participants, individuals who had not fasted for 8 h prior to blood sampling (*n* = 88) and those with missing data on waist circumference, BP, fasting plasma glucose (FPG), TG, and/or HDL-C (*n* = 15) were excluded. Following these exclusions, 1730 premenopausal women were included in the final analysis.

Dietary folate intake and serum levels of the other B vitamins are potential confounding factors, which may be significantly related to serum folate level and resultant MetS. However, the KNHANES dataset did not include information on the dietary folate intake and serum levels of the other B vitamins. Thus, we could not assess the influence of these factors.

During the 2016 to 2018 KNHANES, survey recipients were informed of the random selection of their household for voluntary participation in the nationally-representative survey performed by the KDCA. Written informed consent was obtained before the survey began from all participants who agreed to participate. Blood tests were conducted for subjects aged ≥10 years, and consent was obtained for the blood samples to be used in further studies. The KNHANES data are available on the “Korea National Health and Nutrition Examination Survey” website (https://knhanes.kdca.go.kr, accessed on 31 January 2022). This research was approved by the Institutional Review Board of Yonsei University Gangnam Severance Hospital (Institutional Review Board number: 3-2021-0466). Furthermore, this research was conducted in accordance with the ethical principles of the Declaration of Helsinki.

### 2.2. Measurement of Anthropometric and Laboratory Data

Anthropometric measurements were performed by trained medical staff in accordance with the standardized procedures. With participants wearing light indoor clothing and no shoes, height and weight were gauged to the nearest 0.1 kg and 0.1 cm, respectively. BMI was calculated as weight (kg) divided by height squared (m^2^). BP was gauged three times at 5-min intervals. The means of the second and third measurements were used in the analysis.

Blood samples were collected from the antecubital vein. Serum folate levels were obtained using a chemiluminescent microparticle immunoassay (ARCHITECT i4000SR; ABBOTT, Chicago, IL, USA). FPG, total cholesterol, TG, and HDL-C levels were determined using an automatic analyzer (Hitachi Automatic Analyzer 7600-210, Hitachi, Tokyo, Japan). Leukocyte counts were assessed using an automated blood cell counter (XN-9000; Sysmex, Kobe, Japan).

### 2.3. Definitions of Terms

We defined MetS and its components according to the criteria suggested by the Korean Society for the Study of Obesity and the National Cholesterol Education Program Adult Treatment Panel III [35,36]. Concretely, we defined it as indicating three or more of the following: (1) abdominal obesity (waist circumference ≥85 cm); (2) elevated BP (systolic BP ≥130 mmHg or diastolic BP ≥85 mmHg, or the use of antihypertensives); (3) high FPG (≥100 mg/dL) or use of insulin or diabetes medication; (4) high TG (≥150 mg/dL); and (5) low HDL-C (<50 mg/dL).

Current smokers were persons who had smoked cigarettes during the survey period, and alcohol drinkers were individuals who had consumed alcoholic beverages at least twice a week. Moderate physical activity for ≥30 min for 5 days/week or vigorous physical activity for ≥20 min for 3 days/week were construed as regular exercise.

### 2.4. Statistical Analysis

Sampling weights were utilized to account for the KNHANES complex design. Accordingly, we obtained valid and representative unbiased estimates of all premenopausal Korean women. In KNHANES, the sampling units comprised of households selected using a stratified, multi-stage, probability-sampling design, based on the age, sex, and geographic location. The sample weights were determined to be representative of the South Korean population by taking into cognizance the complex survey design, post-stratification, and non-response. Thus, in the statistical analysis, this study administered sampling weights to account for the complex sampling. Due to the characteristics of KNHANES, the results were reported as standard errors rather than standard deviations.

Serum folate level tertiles were categorized as follows: T1, ≤5.6 ng/mL; T2, 5.7–8.6 ng/mL; T3, ≥8.7 ng/mL. The participants’ characteristics according to serum folate level tertiles were compared using weighted chi-square test and weighted one-way analysis of variance (ANOVA) for categorical and continuous variables, respectively. The odds ratios (ORs) and 95% confidence intervals (CIs) for MetS, abdominal obesity, elevated BP, high FPG, high TG, and low HDL-C were calculated using multiple logistic regression analyses after controlling for potential confounding factors across serum folate level tertiles. All statistical analyses were conducted using IBM SPSS Statistics for Windows, version 25.0 (IBM Corp., Armonk, NY, USA). Statistical significance was set at *p* < 0.05.

## 3. Results

Table 1 shows the participants characteristics according to the tertiles of serum folate levels. The mean values of BMI, waist circumference, and TG tended to decrease proportionally with the increasing folate level tertiles, and the mean values of HDL-C tended to increase proportionally with the increasing folate level tertiles. Additionally, the mean leukocyte count tended to decrease proportionally with serum folate level tertiles.

Table 2 presents the prevalence of MetS and each of its components according to tertiles of serum folate levels. MetS prevalence decreased with increasing folic acid level tertiles as follows: 14.9, 11.0, and 8.6%. Moreover, the prevalence of most MetS components showed trends according to tertiles of serum folate levels. The prevalence of abdominal obesity and high TG decreased with increasing folic acid level tertiles, and the prevalence of low HDL-C increased with increasing folic acid level tertiles.

Table 3 shows the prevalence risk of MetS and its components according to the tertiles of serum folic acid levels. Compared with participants in the highest folic acid level tertile, those in the lowest tertile had significantly higher odds of MetS, abdominal obesity, elevated BP, high TG, and low HDL-C. The multivariate-adjusted ORs (95% CIs) for MetS, abdominal obesity, elevated BP, high TG, and low HDL-C in the highest folic acid level tertile were 2.17 (1.46–3.22), 1.80 (1.25–2.60), 1.77 (1.16–2.70), 1.90 (1.35–2.67), and 1.49 (1.14–1.94) after controlling for age, smoking, alcohol consumption, exercise, residential area, household income, and education level. However, the ORs for high FPG did not show significant differences according to serum folate level tertiles.

## 4. Discussion

This study examined the association of serum folate levels with MetS and its components in a nationally-representative sample of premenopausal Korean women. As this cross-sectional study showed, serum folate levels were independently and inversely associated with MetS and its components (except high FPG) in premenopausal women after adjusting for potential confounding variables. Interestingly, serum folate levels did not show association with high FPG levels. Akbari et al. revealed that folate supplementation significantly lowered the homeostasis model assessment of insulin resistance, but did not affect FPG and HbA1c levels in a randomized controlled meta-analysis [37]. This previous meta-analysis indicated that folate does not influence FPG, but improves insulin resistance by decreasing insulin levels. Our results, showing no association between serum folate and FPG levels, are consistent with the findings of this previous meta-analysis by Akbari et al. A recent study of children and adolescents revealed significantly decreased folate levels observed in groups with obesity and MetS [38]. Moreover, a recent study of older adults suggested that higher folate intake may be related to a lower MetS score [32]. In addition, previous studies have shown that folate levels are associated with metabolic parameters in postmenopausal women. Mahabir et al. revealed that increased BMI, absolute amount of fat, and percent body fat were significantly related to lower serum folate levels in postmenopausal women [33]. Furthermore, Cagnacii et al. reported that high-dose folate administration decreased BP and oxidative stress in postmenopausal women [39,40]. Although the results from these studies indicate a relationship between serum folate levels and unfavorable metabolic parameters, they were confined to postmenopausal women only; therefore, further research is required to demonstrate this relationship in women of reproductive age. Findings of this study are in agreement with the findings of previous research, showing an association between lower serum folate levels and poor metabolic parameters. Moreover, our results suggest that this association is applicable to premenopausal women. Thus, our results expand on earlier findings regarding the relationship between serum folate levels and metabolic parameters. We believe that this is the first large population-based study to examine the association between serum folate levels and MetS and its components in premenopausal women.

Lifestyle behaviors, such as smoking, alcohol consumption, and exercise affect MetS and its components [41,42,43]. Socioeconomic status also influences MetS. Previous studies have indicated that household income and education level are related to the risk of MetS [44,45]. In this study, we included lifestyle and socioeconomic status as confounders in multiple regression analyses to adjust for potential confounding factors.

Several possible mechanisms may underlie the significant inverse association between serum folate levels and MetS and its components. High circulating homocysteine levels in the blood can damage the cardiovascular endothelium and smooth muscle cells, causing endothelial dysfunction and atherosclerosis [46]. Folate is one of the most essential components of homocysteine metabolism and includes the conversion of homocysteine to methionine. Absorbed folate, consumed under normal dietary conditions, is metabolized to 5-methyltetrahydrofolate. A methyl group is provided by 5-methyltetrahydrofolate to convert homocysteine to methionine [47,48]. Therefore, folate deficiency can cause hyperhomocysteinemia [49]. However, the conversion of homocysteine to methionine is not the only possible mechanism. Studies have suggested that folate could directly improve endothelial function by increasing nitric oxide synthesis and bioavailability, independent of its homocysteine-lowering effect [50]. Another important factor to consider is that methyl donors such as folate can reduce systemic inflammation and oxidative stress [51,52]. MetS and related insulin resistance are increasingly recognized as chronic low-grade inflammation [53,54]. Indeed, in the present study, leukocyte count, widely considered a marker of inflammation, tended to decrease proportionally with an increase in serum folate level tertiles. Studies have indicated that folate concentration can systemically influence the levels of inflammation and oxidative stress [55,56]. In addition, the actions of folate in DNA methylation, synthesis, and repair processes can affect the inflammatory phenotype by modulating cell proliferation and epigenetic changes [57]. Based on this evidence, the association between serum folate levels and MetS and its components could be elucidated by oxidative stress and chronic low-grade inflammation.

This study has several limitations that are relevant when interpreting the findings. First, this study did not assess any other potential confounding factors, which may be significantly associated with serum folate level and resultant MetS, including dietary folate intake and serum levels of the other B vitamins. However, since the information on the dietary folate intake and serum levels of the other B vitamins was not included in the KNHANES dataset, we could not assess the influence of these potential confounding factors. Further studies that consider other potential confounding factors including dietary folate intake and serum levels of the other B vitamins are needed. Second, the cross-sectional study design suggests the need for caution with causal and temporal interpretations, and further longitudinal studies are necessary to establish the causality of serum folate levels with MetS and its components in premenopausal women. Third, despite the large sample size, the present study was conducted only in premenopausal Korean women. Thus, our findings may not be generalizable to other ethnic and racial populations. Lastly, this study may not have included several residual confounders related to MetS and used several variables from the self-reporting survey. Despite the potential limitations, this study used a nationally-representative sample of Korean premenopausal women, which supports the general applicability of the present findings. A large sample of study participants allows for an appropriate empirical study of the association of serum folate levels with MetS and its components and strengthens the validity of the findings. Furthermore, a wide range of confounders closely linked with MetS, including lifestyle behaviors and socioeconomic status, were adjusted for in the multiple logistic regression analyses. Additionally, to determine the true nature of serum folate and MetS and its components in premenopausal women, this study excluded participants who underwent hysterectomy and/or oophorectomy, as well as postmenopausal women.

## 5. Conclusions

Serum folate levels showed an inverse association with an increased risk of MetS and most of its components in premenopausal Korean women. These research findings contribute to our understanding of the association between serum folate level and metabolic parameters. The current findings suggest that MetS can be prevented and managed by improving the serum folate levels in premenopausal women.

## Figures and Tables

**Table 1:** Characteristics of the study participants according to serum folate level tertiles.

	All	T1 (≤5.6 ng/mL)	T2 (5.7–8.6 ng/mL)	T3 (≥8.7 ng/mL)	*p*-Value
Unweighted *N*	1730	571	586	573	
Age (years)	35.9 (0.3)	33.6 (0.5)	36.6 (0.5)	37.6 (0.4)	<0.001
BMI (kg/m^2^)	22.7 (0.1)	23.0 (0.2)	22.7 (0.2)	22.2 (0.1)	0.001
Waist circumference (cm)	75.4 (0.3)	75.9 (0.5)	75.7 (0.4)	74.4 (0.4)	0.023
SBP (mmHg)	108.3 (0.4)	108.7 (0.7)	108.5 (0.6)	107.8 (0.6)	0.444
DBP (mmHg)	72.4 (0.3)	72.4 (0.4)	72.5 (0.4)	72.2 (0.4)	0.773
FBG (mg/dL)	92.4 (0.4)	91.6 (0.6)	93.1 (0.7)	92.5 (0.9)	0.311
Total cholesterol (mg/dL)	189.2 (0.9)	186.9 (1.6)	189.9 (1.7)	190.8 (1.5)	0.169
TG (mg/dL)	96.2 (1.6)	105.4 (2.9)	93.1 (2.7)	89.8 (2.3)	<0.001
HDL-C (mg/dL)	57.1 (0.4)	55.6 (0.6)	57.5 (0.6)	58.3 (0.5)	0.001
Leukocyte count (cells/μL)	6060 (48)	6390 (86)	5930 (76)	5840 (77)	<0.001
Current smoker (%)	7.2 (0.7)	10.3 (1.4)	6.6 (1.3)	4.4. (1.0)	0.004
Alcohol drinker (%)	16.6 (0.9)	15.6 (1.6)	17.8 (1.9)	16.6 (1.9)	0.698
Regular exerciser (%)	13.6 (1.0)	10.6 (1.6)	14.3 (1.9)	16.1 (1.8)	0.084
Residence in rural area (%)	11.6 (1.7)	11.8 (2.0)	11.5 (2.2)	11.5 (2.1)	0.981
Household income (US $/month)	4947 (109)	4257 (142)	4684 (169)	4557 (126)	0.100
Education level					0.001
≤Middle school	4.6 (0.6)	6.8 (1.2)	3.4 (0.8)	3.4 (0.9)	
High school	37.3 (1.4)	40.9 (2.3)	38.8 (2.1)	31.9 (2.2)	
≥University	58.1 (1.4)	52.3 (2.5)	57.9 (2.2)	64.7 (2.2)	
Hypertension (%)	3.1 (0.5)	2.8 (0.9)	3.2 (0.8)	3.2 (0.7)	0.931
Diabetes mellitus (%)	1.1 (0.2)	0.6 (0.3)	1.2 (0.4)	1.5 (0.6)	0.313

Data are shown as percentage (standard error) or mean (standard error). *p*-values were calculated by using weighted chi-squared test and one-way ANOVA for categorical and continuous variables, respectively. 1 US $ = 1000 Korean won. BMI, body mass index; SBP, systolic blood pressure; DBP, diastolic blood pressure; FPG, fasting plasma glucose; TG, triglycerides; HDL-C, high-density lipoprotein cholesterol; T1, tertile 1; T2, tertile 2; T3, tertile 3.

**Table 2:** Prevalence of metabolic syndrome and its components according to serum folate level tertiles.

	All	T1 (≤5.6 ng/mL)	T2 (5.7–8.6 ng/mL)	T3 (≥8.7 ng/mL)	*p*-Value
Metabolic syndrome (%)	11.6 (0.8)	14.9 (1.7)	11.0 (1.3)	8.6 (1.2)	0.007
Abdominal obesity (%)	15.1 (1.1)	17.8 (1.8)	16.0 (1.8)	11.4 (1.4)	0.021
Elevated BP (%)	12.7 (0.9)	14.3 (1.6)	12.0 (1.4)	11.7 (1.4)	0.391
High FPG (%)	13.5 (0.9)	11.9 (1.5)	15.8 (1.6)	13.0 (1.6)	0.202
High TG (%)	14.2 (0.9)	17.5 (1.6)	14.0 (1.5)	11.1 (1.2)	0.007
Low HDL-C (%)	45.9 (1.4)	50.3 (2.3)	44.6 (2.3)	42.5 (2.1)	0.033

Data are shown as percentage (standard error). *p*-values were calculated by using weighted chi-squared test. BP, blood pressure; FPG, fasting plasma glucose; TG, triglycerides; HDL-C, high-density lipoprotein cholesterol; T1, tertile 1; T2, tertile 2; T3, tertile 3.

**Table 3:** Multivariate-adjusted odds ratios and 95% confidence intervals for metabolic syndrome and its components according to serum folate level tertiles.

	T1 (≤5.6 ng/mL)	T2 (5.7–8.6 ng/mL)	T3 (≥8.7 ng/mL)
Metabolic syndrome (%)	2.17 (1.46–3.22)	1.35 (0.89–2.05)	1
Abdominal obesity (%)	1.80 (1.25–2.60)	1.51 (1.04–2.21)	1
Elevated BP (%)	1.77 (1.16–2.70)	1.04 (0.69–1.58)	1
High FPG (%)	0.91 (0.62–1.33)	1.25 (0.87–1.81)	1
High TG (%)	1.90 (1.35–2.67)	1.34 (0.95–1.90)	1
Low HDL-C (%)	1.49 (1.14–1.94)	1.15 (0.88–1.49)	1

Odds ratios for metabolic syndrome, abdominal obesity, elevated EP, high FPG, high TG, and low HDL-C were determined by using multiple logistic regression analysis after adjusting for age, smoking, alcohol consumption, exercise, residential area, household income, and education level. BP, blood pressure; FPG, fasting plasma glucose; TG, triglycerides; HDL-C, high-density lipoprotein cholesterol; T1, tertile 1; T2, tertile 2; T3, tertile 3.

## Data Availability

Publicly available datasets were analyzed in this study. The data can be found on the official website of the Korea Disease Control and Prevention Agency (https://knhanes.kdca.go.kr/knhanes/sub03/sub03_02_05.do; accessed on 31 January 2022).
